# Mechanical properties of emulsified recycled cement-stabilized macadam based on step-by-step filling gradation design

**DOI:** 10.1371/journal.pone.0268105

**Published:** 2022-05-11

**Authors:** Zhijun Liu, Chenhui Li, Tao Sun, Liangliang Wang

**Affiliations:** State Key Laboratory for Geomechanics and Deep Underground Engineering, School of Mechanics & Civil Engineering, China University of Mining & Technology, Xuzhou, China; Beijing University of Technology, CHINA

## Abstract

Since the recycling of waste original cement-stabilized macadam (OCSM) base has important environmental and economic significance, the addition of emulsified asphalt to OCSM to form emulsified recycled OCSM (ER-OCSM) can improve the flexibility of recycled mixtures. However, the influence of emulsified asphalt on the mechanical performance of such mixtures remains to be investigated. This study presents a gradation design and ER-OCSM established using the step-by-step filling method and investigated the mechanical properties of the ER-OCSM mixture. The apparent characteristics, crushing value and needle-like particle content of the OCSM milling material were tested. Based on step-by-step filling theory, the appropriate test method to achieve a uniform and dense state according to the characteristics of different aggregates was selected, and the dense skeleton gradation design method for recycled cement macadam was obtained. The mechanical properties of the ER-OCSM were analyzed by performing indoor physical laboratory tests. The natural gradation of the OCSM milling material exceeded the gradation range recommended in the Technical Guide for the Promotion of Science and Technology of the Construction Project of the Main Highway in Jiangsu Province (Trial), but the designed gradations were basically within the range. At the same age and temperature, the flexural strength and dynamic elastic modulus of the ER-OCSM decreased gradually with an increase in the emulsified asphalt content. Because ER-OCSM had temperature-sensitive characteristics, the adhesiveness of the asphalt between particles in the mixture decreased with increasing temperature, which was manifested as the unconfined compressive strength, flexural tensile strength and dynamic elastic modulus decreasing with an increase in temperature (the decrease was slight within 5–25°C but noticeable within 25–60°C). Furthermore, a higher emulsified asphalt content caused a more noticeable decrease. The flexural strength of the tested ER-OCSM showed noticeable correlations with the splitting strength, unconfined compressive strength and dynamic elastic modulus. The proper addition of emulsified asphalt can reduce the rigidity of ER-OCSM. However, the emulsified asphalt content should be strictly controlled; otherwise, the mechanical properties of the material will decrease greatly, adversely impacting the comprehensive road use performance.

## Introduction

Cement-stabilized macadam has a number of advantages, such as a high stiffness, high strength, and satisfactory water stability. It is widely used in the base of high-grade highways. In the large- and medium-scale modifications of high-grade highways, the original cement-stabilized macadam (OCSM) base is often replaced with new materials to meet performance requirements. If the OCSM base can be recycled, resources can be saved, the environment can be protected, and the project cost can be reduced. Therefore, research on OCSM recycling has become a popular topic in transportation science and technology research [1–7]. For recycled cement-stabilized macadam, aggregate gradation and admixtures are two important factors that affect road performance.

Studies have shown that the use of a dense skeleton gradation can improve the mechanical properties and crack resistance of cement-stabilized macadam [8–11]. The standard [12] recommends the use of emulsified asphalt and a certain proportion of cement for recycling waste asphalt pavement. However, due to the different characteristics of recycled aggregates and the different contents of OCSM in recycled mixtures, the performances of the final mixtures are inevitably different. Cai et al. [3] found that recycled aggregates have the characteristics of a high water absorption and low compressive strength and that recycled materials are prone to shrinkage cracking. According to Ren et al. [13], the content and composition of recycled aggregates have a substantial effect on the mechanical properties of cement-stabilized cold recycled mixtures. Yan et al. [14] investigated cement-emulsified asphalt recycled mixtures and found that the optimal aggregate gradation should be designed before the optimal contents of emulsified asphalt and cement are determined.

Emulsified asphalt and cement are commonly used as admixtures in highway materials. In the 1960s, Terrel et al. [15] and Head [16] proposed the method of using cement to treat emulsified asphalt mixtures and found that the early strength of a mixture could be improved with this method, which accelerated the road construction process and allowed the road to be open to traffic earlier. James et al. [17] added a small amount of cement to emulsified asphalt mixtures, and the strength and stiffness of the mixtures were greatly improved. According to engineering practice in South Africa, Bullen et al. [18] found that if the amount of cement in emulsified asphalt mixtures is too large, fatigue cracking and transverse cracking are likely to occur. Li et al. [19] carried out indoor tests on the fatigue performance, strength, stiffness, temperature sensitivity and stress–strain relationship of cement-emulsified asphalt mixtures. The test results showed that these mixtures had a longer fatigue life, as the pure cement concrete had a lower temperature sensitivity and the pure asphalt concrete had a higher flexibility. Unger et al. [20] mixed emulsified asphalt as a recycling agent with 100% reclaimed asphalt pavement (RAP) to form a new test section of pavement base, used a falling weight deflectometer (FWD) to detect its structure, and concluded that the method of using emulsified asphalt to regenerate RAP asphalt mixtures is relatively reliable. Pi et al. [21] investigated the strength formation mechanism of emulsified asphalt cold recycled mixtures and analyzed the influences of the gradation, emulsified asphalt content, cement content and other factors on the performance of the cold recycled mixtures; the optimal amounts of emulsified asphalt and cement in RAP material were determined through testing. When cement is used as the regeneration agent of a recycled mixture base, higher cement contents can effectively improve the compressive strength of the mixture [22,23]. Recasens et al. [24] investigated recycled mixtures using emulsified asphalt and cement as the recycling agents and found that the addition of emulsified asphalt and cement could improve the mechanical properties of the mixtures, which increased the flexibility of the mixtures and improved their crack resistance. Despite a number of studies on regeneration asphalt pavement materials with emulsified asphalt and cement, most focused on the mixture of waste asphalt pavement materials with base materials, asphalt pavement materials, and the mixture of waste asphalt pavement materials with recycled base materials and some new materials [25–29]. Currently, systemic research on the regeneration of OCSM with cement and emulsified asphalt remains to be carried out.

Overall, the addition of emulsified asphalt can improve the flexibility, enhance the crack resistance, and affect the mechanical properties of a recycled mixture. Based on previous research, gradations of emulsified recycled OCSM (ER-OCSM) were designed using the step-by-step filling method, and the mechanical properties of ER-OCSM mixtures were investigated. To achieve these goals, the properties of the raw ER-OCSM materials were tested, and the aggregation gradation method was designed. With unconfined compressive strength as the control index, the most appropriate cement content of the ER-OCSM mixture was determined. Then, the influences of the emulsified asphalt content, age and temperature on the mechanical properties of the ER-OCSM, as well as the underlying mechanisms, were analyzed. The results of this study may lay a foundation for the technical application of ER-OCSM and provide a reference for research on similar recycled cement-stabilized materials.

## Materials and methods

### Raw materials

The cement used for the test was ordinary silicate cement "PO42.5" (Hanbang "PO42.5", Huaihai Cement Plant, Xuzhou, China). The main technical indices are summarized in Tables 1 and 2.

**Table 1:** Technical indices of the cement used in this study.

Item	Stability/mm	Setting time/min		Compressive strength/MPa		Flexural strength/MPa	
		Initial setting time	Final setting time	3 d	28 d	3 d	28 d
Requirement	≤5	≥45	≤600	≥26±2.0	≥42.5	≥4	≥6.5
Measured value	1.1	186	321	26.1	45.2	4.7	7.6

The emulsified asphalt used in this test was cationic medium-breaking emulsified asphalt (Xuzhou Luxin Highway Materials Co., Ltd., Xuzhou, China).

**Table 2:** Technical indices of the emulsified asphalt.

Project	Demulsification rate	Particle charge	Screen residue (1.18 mm)/%	Evaporation residue				Storage stability at room temperature	
				Content/%	Penetration (25°C)/0.1 mm	Softening point/°C	Ductility (5°C)/cm	1 d/%	5 d/%
Experimental value	Medium breaking	+	0.06	56.7	89	59.0	42.3	0.5	2.6
Technical requirement	Medium breaking	+	≤0.1	≥53	80–130	≥50	≥30	≤1	≤5

In this study, OCSM milling material was used as the recycled aggregate, which was taken from the milling engineering site of a cement-stabilized macadam base of a first-class highway in Jiangsu Province.

The water used in this study was standard drinking water.

### Analysis of the surface characteristics of the recycled aggregates

**Fig 1:**
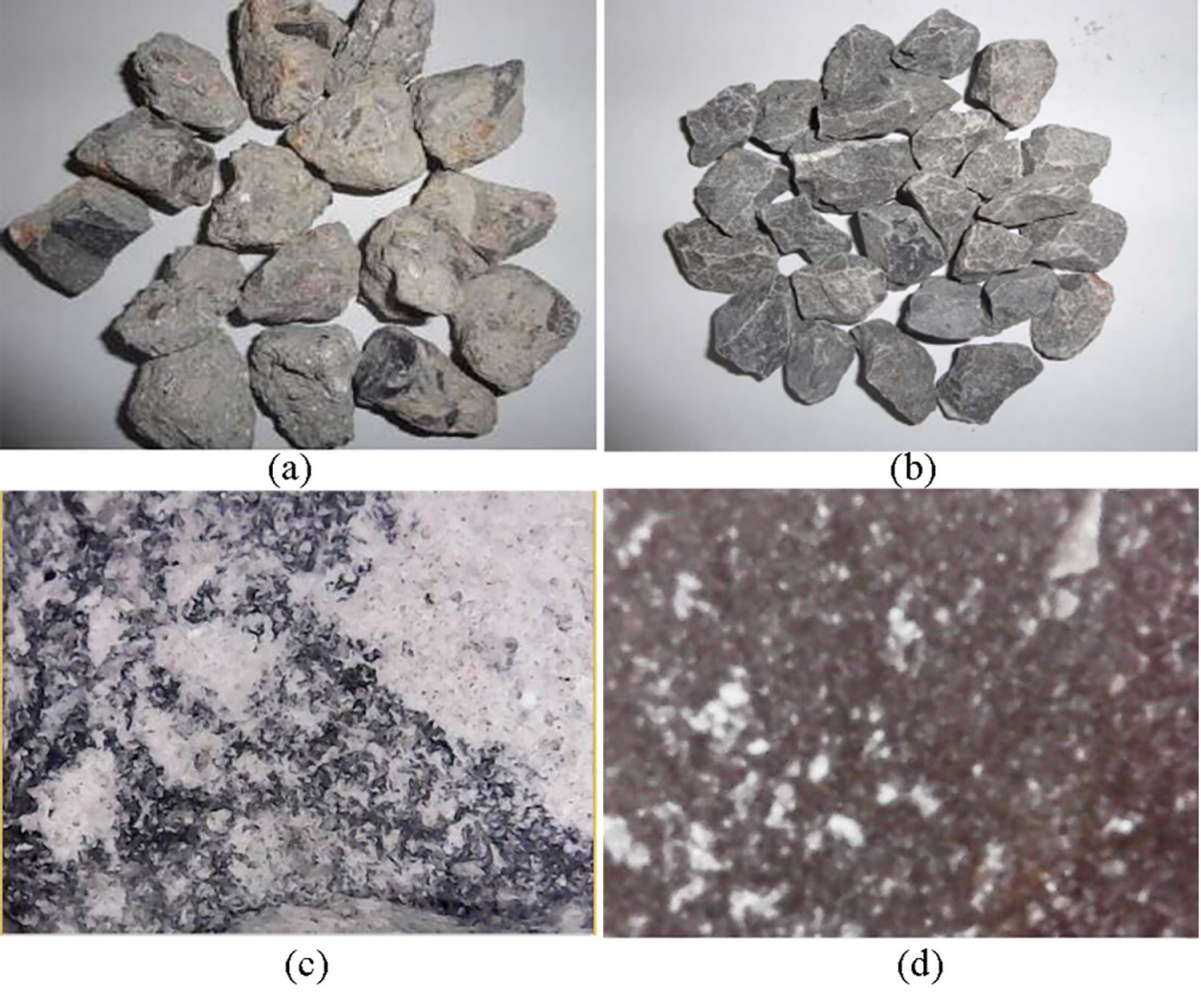
Recycled aggregates and common aggregates. (a) Recycled aggregates. (b) Common aggregates. (c) Recycled aggregates (magnitude, 30 ×). (d) Common aggregates (magnitude, 30-fold).

First, the adopted recycled aggregates and common aggregates were observed (Fig 1: coarse aggregates), and a comparison suggests that the recycled aggregates had the following surface characteristics:

1. Most of the recycled coarse aggregates (≥ 4.75 mm) were stones encased in cement mortar, a small part was cement mortar with a few aggregated stones, and a very small part was cement mortar particles.
2. The recycled fine aggregates (≤ 4.75 mm) were divided into three categories: stone chips with cement mortar on the surface, stone chips without cement mortar on the surface, and stone chip particles and stone powder produced in the process of crushing.
3. The amount of cement mortar around the crushed stone with different particle sizes varied.
  The surface area of the cement mortar that covered the coarse aggregates was large. Some of the original crushed stones were completely encased, and some were partially covered or exhibited a honeycomb-like appearance.
4. The surfaces of the natural aggregate particles were clean, smooth and glossy, with few voids. In contrast, the surfaces of the recycled aggregate particles were not clean; they were dark, and the particles at the edges and corners were round. Cement mortar, which was rough due to pores and cracks, was stuck to the surfaces of the particles.

This comparison showed that the surfaces of the recycled aggregates were rougher than those of the common aggregates. The surfaces of the recycled aggregates were encased in cement mortar, which increased the specific surface area of the aggregate particles. These characteristics enhanced the adsorption capacity of the aggregates and the cohesion of the material. Additionally, small cracks were observed on the surface of the recycled aggregate, which could make it easier for cement particles to enter the recycled aggregates and further enhance the bonding performance of the material.

Three groups of screening tests on recycled aggregates were carried out with a shaker, and two parallel tests were conducted for each group of tests. The results of the screening tests on the recycled aggregates are summarized in Table 3.

**Table 3:** Screening test results.

Gradation	Mass percentage (%) of the material filtered through the following sieve pore (mm)						
	31.5	19	9.5	4.75	2.36	0.6	0.075
Test gradation range	97–100	85–92	66–73	44–49	24–29	9–14	1–3
Recommended gradation range provided by the standards	100	68–86	38–58	22–32	16–28	8–15	0–3

Note: The gradation range recommended by the standards refers to the recommended gradation of the anti-crack and embedded cement-stabilized macadam mixture in the Technical Guide for the Promotion of Science and Technology of the Construction Project of the Main Highway in Jiangsu Province (Construction Technology of the Pavement Base of Anti-crack and Embedded Cement Stabilized Macadam (Trial)).

The screening tests showed that almost no aggregates remained on the screen with a pore size of 31.5 mm, which indicated that for OCSM, the requirements of particle crushing can be satisfied by merely milling (the maximum particle size was less than 31.5 mm, which accounts for more than 97%). The passing rates of the sieves with pore sizes of 19 mm, 9.5 mm and 4.75 mm were much higher than those specified in the standards, which indicates that both the original OCSM base and the aggregates with a particle size of 4.75–19 mm in the graded base were fully broken during the milling and crushing process, and the cement mortar for the adhesion of the mixtures of the base were broken into finer particles as well, which eventually led to increased passing rates through these sieves.

The needle-like particle content of the recycled coarse aggregates was tested, and the results are summarized in Table 4.

**Table 4:** Needle-like particle content of the recycled coarse aggregates.

Particle size (mm)	Above 31.5	31.5–19	19–9.5	9.5–4.75
Needle-like particle content (%)	0.0	10.2	15.3	15.8

Table 4 shows that the needle-like particle content of the recycled coarse aggregates met the requirements of the specification for the needle-like particle content of the base material ≤ 18% under extremely heavy and extra-heavy traffic conditions [30], which indicates that the milling process can produce recycled coarse aggregates with a qualified needle-like particle content.

The crushing value of the recycled coarse aggregates was 27.31%, which was slightly higher than the requirement recommended in the standards that the crushing value of the base material is ≤ 26% under extremely heavy traffic conditions [30]. This result may be because the recycled aggregates, especially the coarse aggregates, had cement mortar and small stones attached to their surfaces, so their resistance to pressure was weak. In the process of gradually increasing the load, finer stone chips or stones easily fell off the aggregates under the effect of intercalation. Therefore, the crushing value obtained in the test was higher than the actual value. This result also indicates that the surface characteristics of recycled aggregates have a certain impact on their physical properties.

### Gradation design of the recycled aggregates

In this study, aggregate gradations were designed based on progressive filling theory. Progressive filling theory is based on filling theory, particle interference theory and maximum density curve theory. It takes intercalation as the principle and focuses on producing a dense gradation with a minimum void ratio in the mixture. According to particle interference theory, if the maximum compactness of a mixture is to be achieved, the voids of the uppermost level of particles should be filled by the particles in the next lower level, the remaining voids should be filled by the particles in the following level, and so on.

However, if the particle size for filling the gap is larger than the gap between the particles, it will lead to the occurrence of interference. Therefore, the size of the filling particles should be distributed according to a certain proportion. According to filling theory, when the aggregates are in the loosest state, the void ratio is 48%. At this moment, the voids between the main aggregates can be filled with smaller particles to obtain a better filling effect compared to that obtained with the next particle size. According to the theory of the maximum density curve, the closer the particle grading curve of the mineral aggregates is to a parabola, the higher the density of the mixture [31–33].

In this study, 4.75 mm was taken as the dividing boundary between coarse and fine aggregates. The aggregates with a particle size greater than 4.75 mm were coarse aggregates, and the aggregates with a particle size less than 4.75 mm were fine aggregates. According to step-by-step filling theory, the bulk density of the dense aggregates was measured to determine the optimal ratio of each particle size of the aggregates to determine the final gradation. The whole test was divided into three parts: the gradation design of the coarse aggregates, the gradation design of the fine aggregates and the composite gradation design of the coarse and fine aggregates. The main goal was to select the appropriate test method based on the characteristics of different aggregates to achieve a uniform and dense state.

#### (1) Coarse aggregate gradation design

The recycled aggregates were divided into three grades: D_1_ (31.5–19 mm), D_2_ (19–9.5 mm) and D_3_ (9.5–4.75 mm). To better simulate the compaction effect of the vibratory roller on road materials at a construction site, the following two-stage filling test was carried out using the vibration compaction method [34].

Grade I filling: D_1_ aggregates were taken as the main aggregates, and the D_2_ aggregates and D_1_ aggregates were mixed according to a ratio of 5:95. The amount of D_2_ was increased by 5% on the basis that the total mass fraction remained constant. A grade I tamping filling test was conducted. The result near the optimal filling aggregate content was selected to construct the curve of the relationship of the filling aggregate content and bulk density of the two grades of aggregates. The wave peak values were compared, and the optimal D_1_:D_2_ ratio corresponding to the highest point was recorded.

Grade II filling: On the basis of the grade I filling compaction test, the optimal D_1_:D_2_ ratio was adopted for the main body of the grade II compaction test, and for the next level, D_3_ and D_1_+D_2_ were mixed at a ratio of 5:95. The amount of D_3_ was increased by 5% on the basis that the total mass fraction remained constant. A grade II tamping filling test was conducted. The relationship curve of the filling aggregate content and the bulk density of the mixture was constructed, and the (D_1_+D_2_):D_3_ ratio corresponding to the maximum density of the relation curve was obtained.

**Fig 2:**
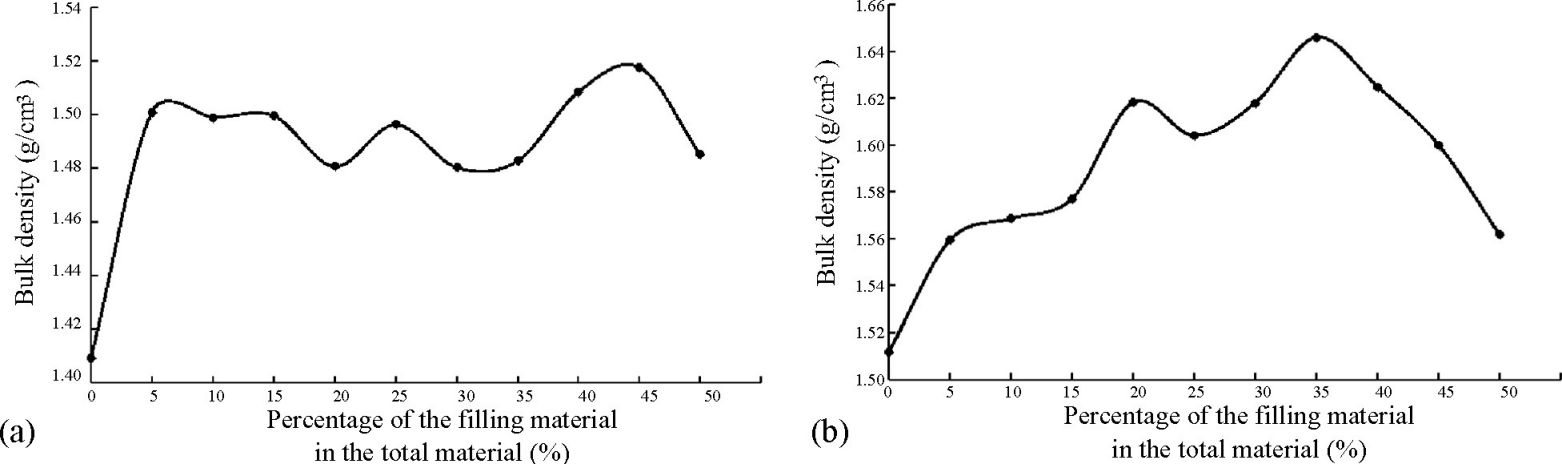
Results of the step-by-step filling test on the coarse aggregates. (a) Grade I filling. (b) Grade II filling.

The results of the step-by-step filling test on the coarse aggregates are shown in Fig 2.

As shown in Fig 2, when D_1_:D_2_ was 55:45 in the grade I filling test, the bulk density of the recycled mixed aggregates reached a maximum. In the grade II filling test, when (D_1_+D_2_):D_3_ was 65:35, the density of the mixed aggregates reached a maximum.

**Fig 3:**
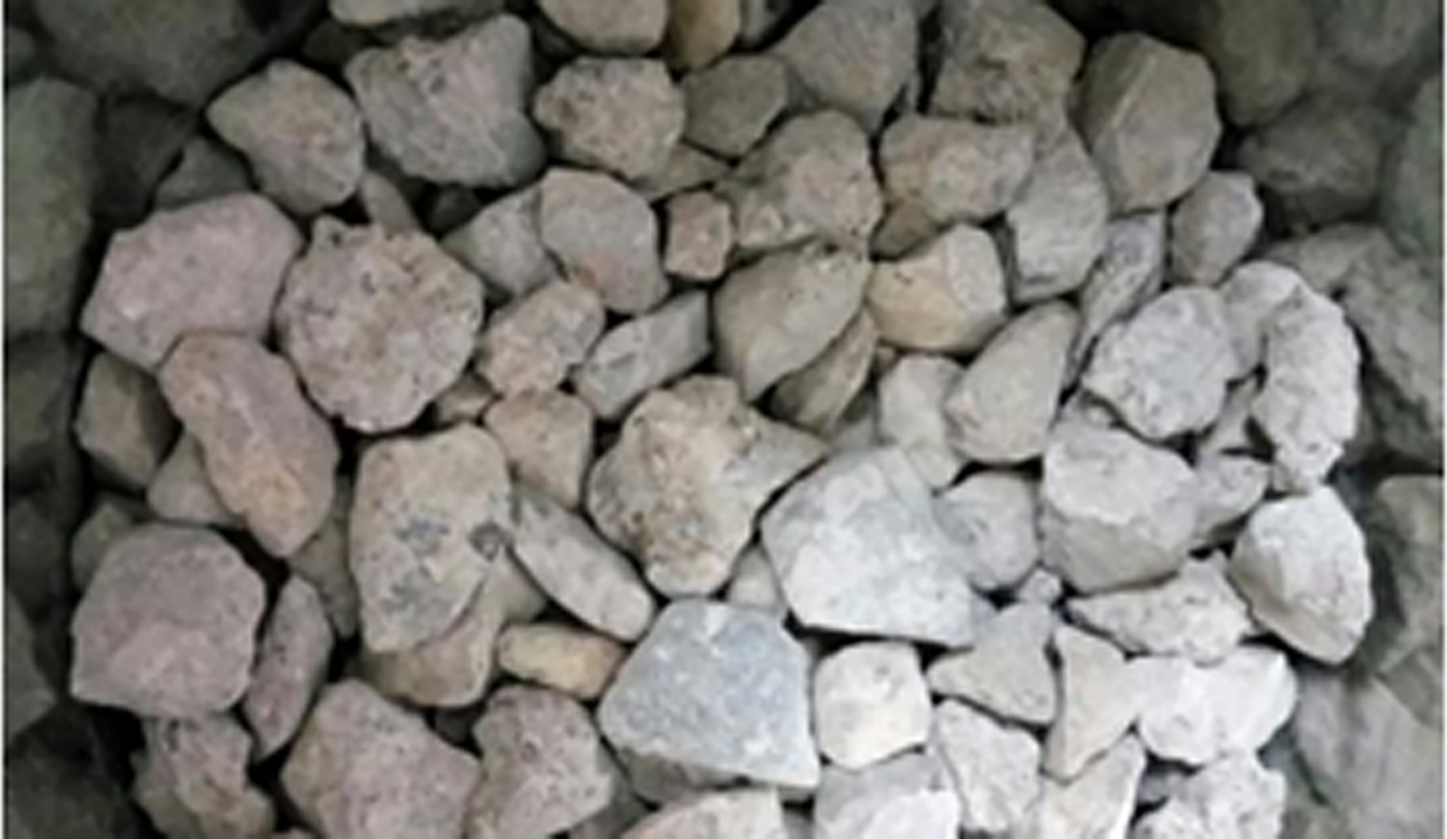
Effect of step-by-step filling and compaction on the coarse aggregates.

According to the results of the two-stage compaction test (D_1_:D_2_ = 55:45 and (D_1_+D_2_):D_3_ = 65:35), when the mixing ratios of the three grades of aggregates were 35.75:29.25:35, the skeleton structure formed by intercalation among the aggregates was the densest (see Fig 3), and the coarse aggregate gradation of the dense skeleton gradation recycled cement-stabilized macadam was obtained, as shown in Table 5.

**Table 5:** Gradation determined by the step-by-step filling test on the coarse aggregates.

Particle diameter/mm	31.5	19	9.5
Passing rate/%	100	64.25	35

#### (2) Gradation design of fine aggregates

**Fig 4:**
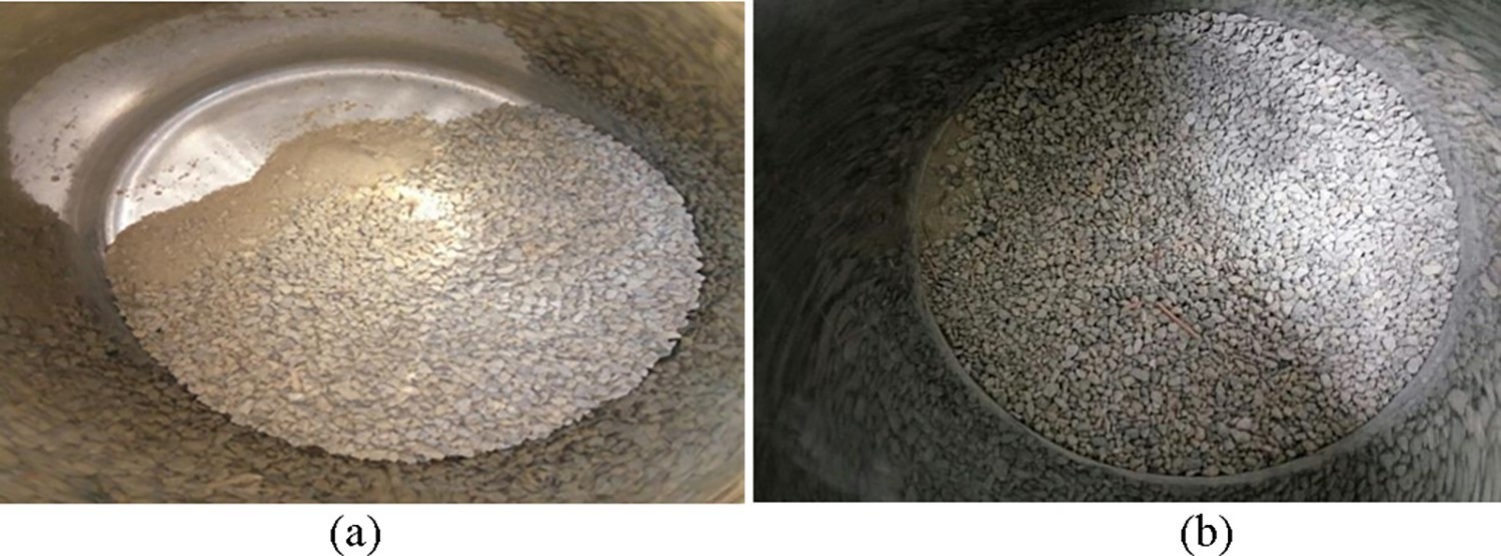
Comparison of the aggregate filling effects. (a) Compaction effect. (b) Tamping effect.

The recycled fine aggregates were divided into four grades: D1 (4.75–2.36 mm), D2 (2.36–0.6 mm), D3 (0.075 mm) and D4 (less than 0.075 mm). In the process of the gradation design of the fine aggregates with the step-by-step filling method, serious particle separation in the process of fine aggregate tamping occurred, while premixed tamping achieved a noticeably better effect (Fig 4). Therefore, the premixed tamping method was used for fine aggregate compaction in this study.

**Fig 5:**
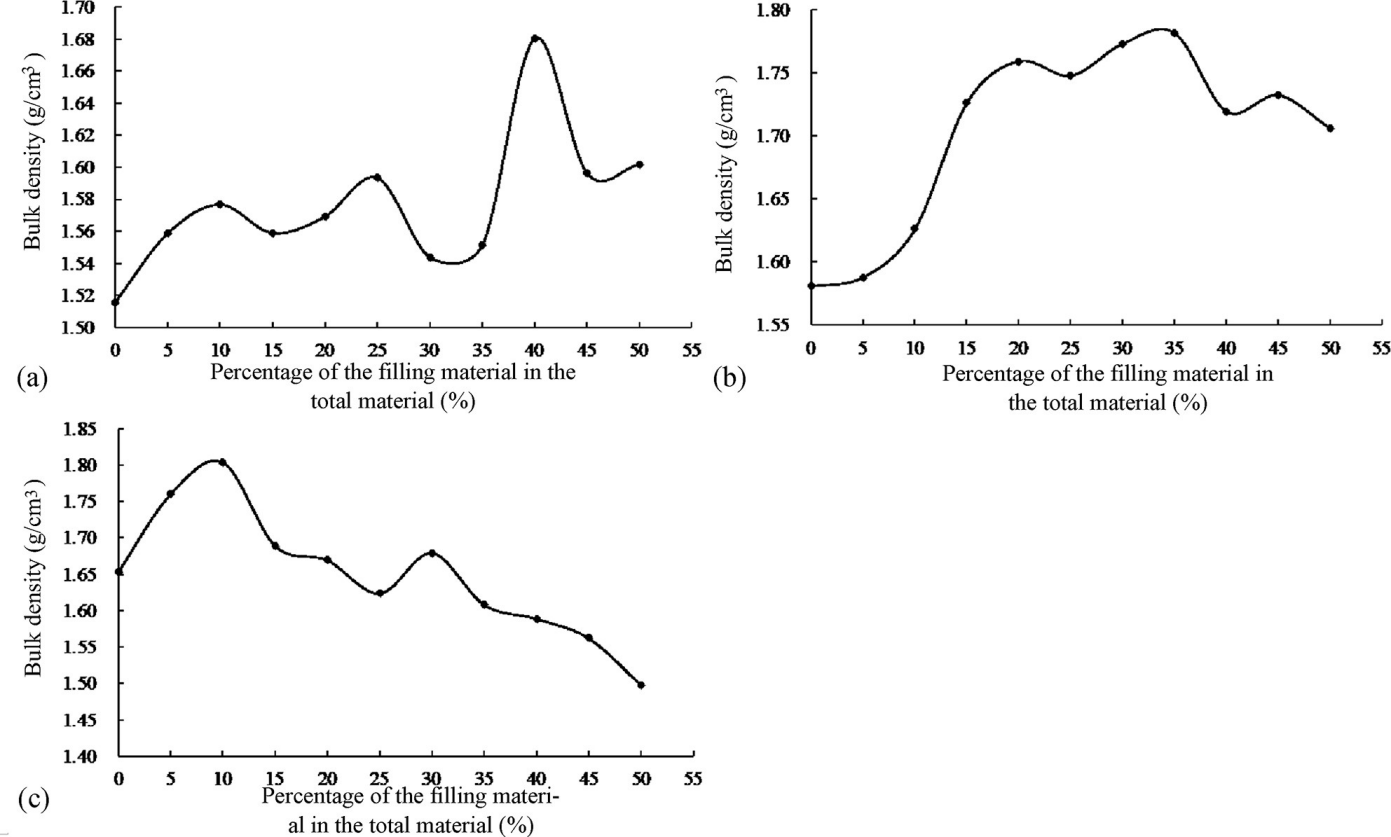
Step-by-step filling of the fine aggregates: (a) Grade III filling. (b) Grade IV filling. (c) Grade V filling.

A total of three filling tests were performed. The specific steps were based on the coarse aggregate gradation design. The test results are shown in Fig 5.

The grade III filling test showed that the bulk density peaked when the ratio of the main aggregates to secondary aggregates was 60:40.

The grade IV filling test showed that the bulk density increased with an increase in the filling aggregate content, and the peak value corresponded to 35%. The optimal ratio of (D_1_+D_2_):D3 was determined to be 65:35.

The results of the grade V filling showed that when the filler content increased, the powder aggregates filled the voids in the mixture, and the void ratio curve first increased and then decreased. The underlying reasons might be as follows. In the early stage of filling, the powder aggregates play a satisfactory role in filling the gaps, and therefore, the voids of the mixture are reduced. Afterward, with the increase in the powder particles, the dense structure of the original main aggregates is destroyed, the mixture presents a suspended dense structure, and the void ratio gradually increases. As the content of the powder particles continues to increase, the powder particles account for the main part of the mixture. Because it is difficult for powder particles to form an effective skeleton structure, the porosity of the mixture is no longer determined by the skeleton structure, and the density presents a gradually decreasing trend. The optimal ratio of (D_1_+D_2_+D_3_):D4 was 90:10. After calculation, the final fine aggregate gradation design was determined, which is shown in Table 6.

**Table 6:** Gradation of the fine aggregates determined by step-by-step filling.

Particle diameter/mm	4.75	2.36	0.6	0.075
Passing rate/%	100	64.9	41.5	10

#### (3) Integrated design of coarse and fine aggregates

**Fig 6:**
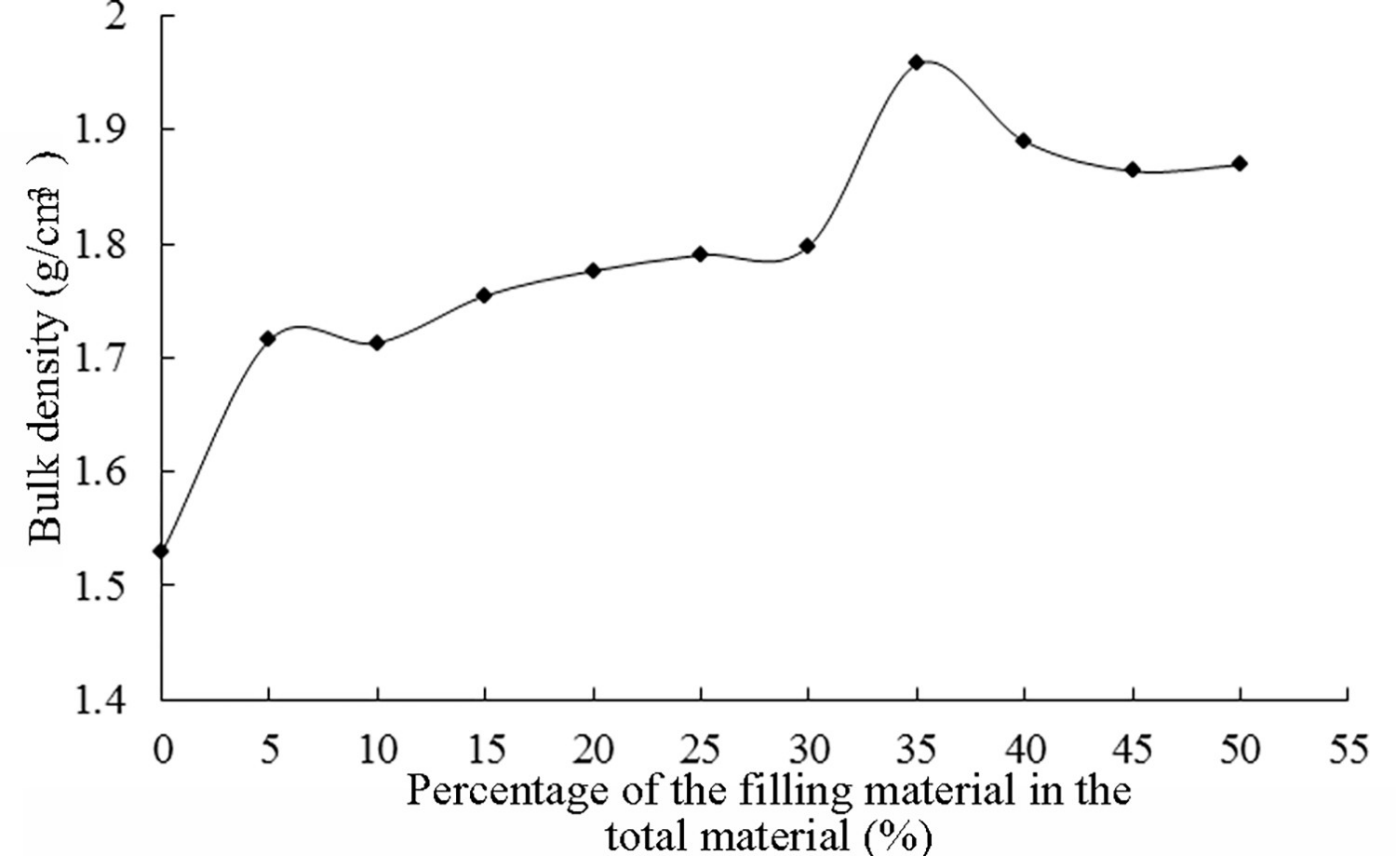
Combined design of the coarse and fine aggregates.

Based on the two parts of the tests described above, the determined coarse and fine aggregate gradations could be closely arranged to form a mixture with a high compactness and satisfactory skeleton structure. To determine the optimal proportion of coarse and fine aggregates, a combined design of coarse and fine aggregates should be established. Thus, the final three-grade proportion of coarse aggregates determined was used to create the main aggregate component of the combined design. The proportion of the fine aggregates was incrementally increased by 5% until the fine aggregates provided the total mass required for the tamping test, and the curve of the relationship between the bulk density and the filling fraction was established, as shown in Fig 6.

Fig 6 shows that the bulk density of the fine aggregates first increased and then decreased with an increase in the filling aggregate content. When the ratio of the coarse aggregates to the fine aggregates was 65:35, the bulk density peaked. This peak value corresponded to the optimal composite gradation ratio of the coarse and fine recycled aggregates.

Finally, the gradation of the recycled aggregates for a dense skeleton was determined (Table 7).

**Table 7:** Gradation of the recycled aggregates.

Particle diameter/mm	31.5	19	9.5	4.75	2.36	0.6	0.075
Experimental composite gradation	100.0	76.8	57.8	35.0	23.6	10.6	2.7
Upper limit of the standard	100	86	58	32	28	15	3
Lower limit of the standard	100	68	38	22	16	8	0

**Fig 7:**
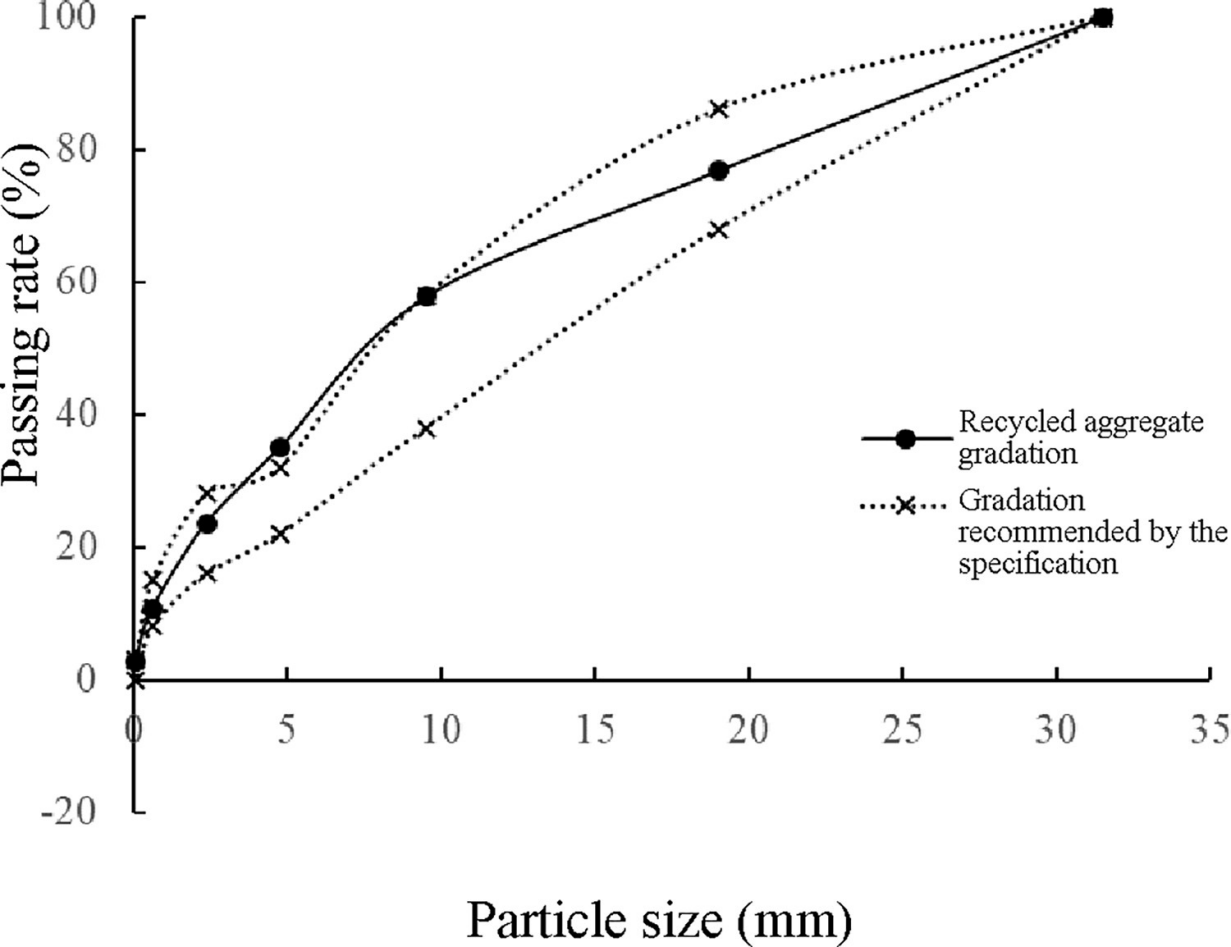
Gradation of the recycled aggregates.

Fig 7 shows that most of the particle size ranges of the recycled aggregate gradation were within the range recommended in the specification [35], and only the particle size range of 9.5–4.75 mm was slightly higher than the upper limit of the specification. Presumably, compared with the aggregates of ordinary cement-stabilized macadam, most of the coarse aggregates in the OCSM granular material were encased in cement mortar, which increased the void space among the aggregate particles. Therefore, more aggregates below 4.75 mm were needed for step-by-step filling, and the passing rate of the particles corresponding to the final recycled aggregate gradation was slightly higher than the upper limit of the specification [35].

## OCSM performance test

### Test design

Specimens were prepared based on the obtained results of the gradation design. The mechanical properties (flexural strength and dynamic elastic modulus) of the ER-OCSM specimens were tested by laboratory physical testing. First, the unconfined compressive strengths of ER-OCSM specimens with different cement contents (2.0%, 3.0%, 3.5%, 4.0%, 4.5%, 5.0%, and 6.0%) but without emulsified asphalt added were obtained [36]; here, cement content refers to the proportion of the mass of the cement to the total mass of the aggregate calculated by the external-adding method [37]. The amounts of cement used here were determined by following the requirement that the unconfined compressive strengths of cement stabilized materials with an age of 7 d should be between 5 MPa and 7 MPa, as presented by the standards under extremely heavy and extra-heavy traffic conditions [30]. Based on the determined cement amount, the mechanical indices of the ER-OCSM specimens under the influence of different emulsified asphalt contents (1.0%, 2.0%, 4.0%, and 6.0%) and different temperatures (5°C, 15°C, 25°C, 40°C, and 60°C) [38] were also determined (Table 8).

**Table 8:** Protocols for the mechanical performance tests.

Testing indices	Age/d	Specimen type	Size/mm	Number of parallel specimens
Unconfined compressive strength	7	Column	150×150	13
Flexural tensile strength Dynamic elastic modulus	90	Beam	400×100×100	12
	7/28/60/90	Beam	400×100×100	12

### Specimen molding mode

#### Specimen material preparation

**Fig 8:**
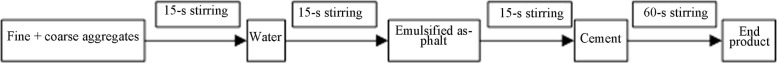
Mixing scheme.

The specimens were prepared according to the gradation and compaction test results. According to the research by Sun et al. [39], the following mixing scheme (Fig 8) can make a mixture reach a highly uniform state. The mixture was evenly mixed and then put into the mold. To prevent premature demulsification of the emulsified asphalt before molding, the specimens loaded into the mold were immediately pressed.

#### Static pressing

The mold and test material were transferred to an electrohydraulic press, and 98% compaction was achieved with static pressing. In the process of static pressing, the loading speed of the electrohydraulic press was controlled. After the die was pressed into the mold, the pressure was maintained for a certain period of time.

#### Demolding

After hydrostatic molding, the column specimens rested at room temperature for 6 h before demolding (if the specimens sat for too long, it would have made the demolding process more difficult, and the demolded specimens could easily be damaged, which would affect specimen integrity). After static pressing, the beam specimens rested at room temperature for 12 h, and then the mold was removed (because the beam specimens were large, early removal of the mold would affect the specimen integrity as well as the subsequent test results).

#### Specimen curing

**Fig 9:**
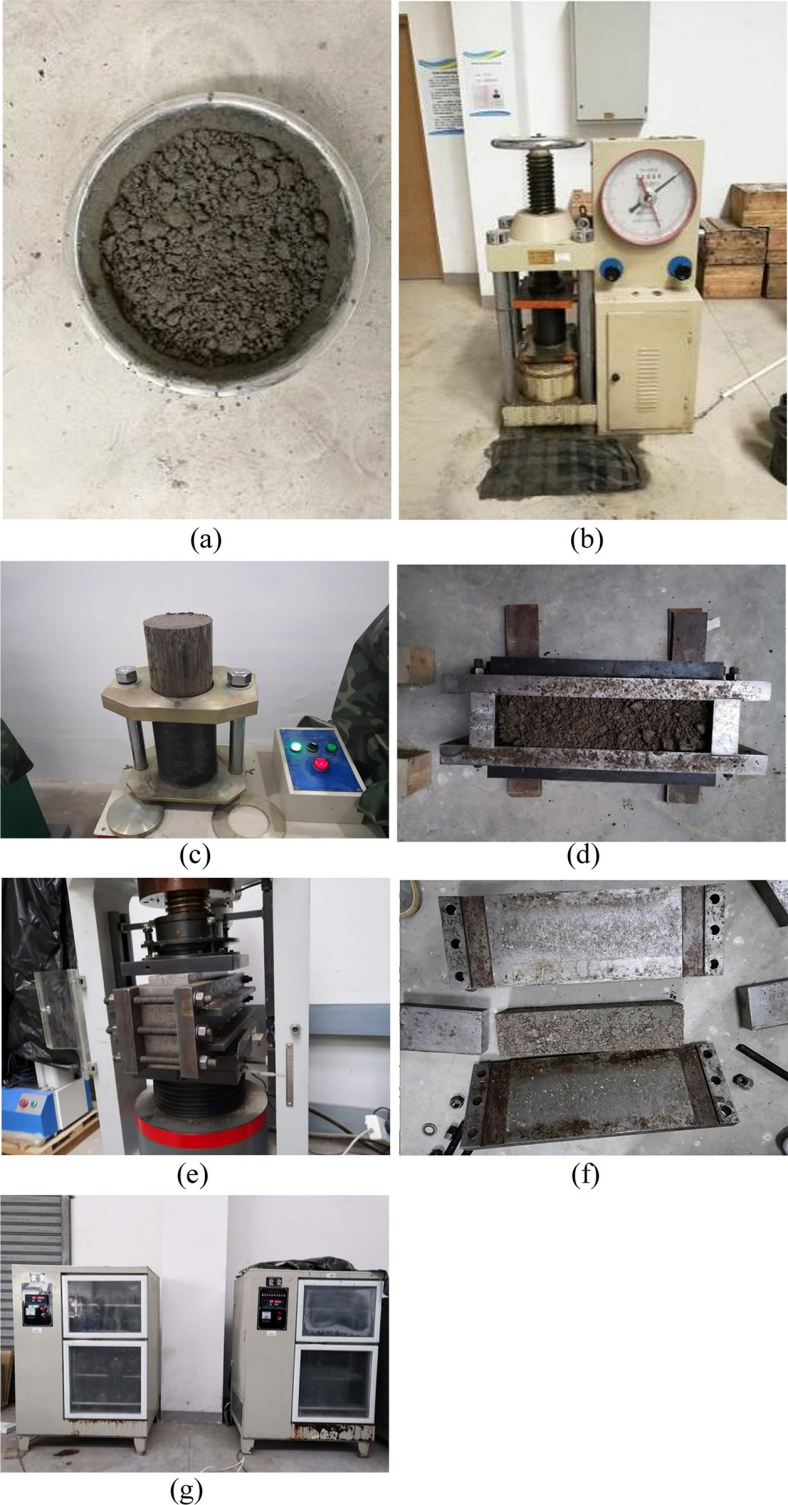
Specimen preparation and curing. (a) Mixing of the ER-OCSM mixtures. (b) Compaction to form column specimens. (c) Demolding of the column specimens. (d) Material loading for beam specimens. (e) Pressing to form beam specimens. (f) Demolding of the beam specimens. (g) Specimen curing (partial setup).

After demolding, the specimens were wrapped in black plastic bags and placed under standard curing conditions (temperature of 20°C± 2°C; relative humidity greater than or equal to 95%). The preparation and curing processes of the specimens are shown in Fig 9.

### Determination of the optimal moisture content and maximum dry density

According to the literature [40], the optimal water contents and maximum dry densities of recycled mixtures with different cement contents under different gradations were obtained (Table 9). Based on 7-d unconfined compressive strength tests, a minimum cement content of 3.5%, which meets the requirements specified in the standards [30], was taken as the cement content for the ER-OCSM (Table 10). Compaction tests were performed, and the optimal water contents and maximum dry densities of recycled mixtures with different emulsified asphalt contents were obtained (Table 11).

**Table 9:** Optimal water contents and maximum dry densities of the mixtures with different cement contents.

Test index	Cement content/%						
	2.0	3.0	3.5	4.0	4.5	5.0	6.0
Optimal water content *ω*_o_/%	7.11	7.27	7.29	7.30	7.48	7.45	7.63
Maximum dry density *ρ*_max_/(g/cm^3^)	2.160	2.170	2.180	2.200	2.220	2.240	2.245

**Table 10:** The 7-d unconfined compressive strengths of the macadam mixtures with different cement contents.

Cement content/%	2	3	3.5	4	4.5	5	6
Unconfined compressive strength/MPa	4.32	5.88	6.88	7.47	7.80	8.16	8.50

Note: Among the 13 parallel specimens of the same group for the unconfined compressive strength test, most abnormal values were eliminated (according to the three-fold mean squared error method, the values with a difference of more than three times the mean squared error compared with the average will be excluded), but one or two abnormal values were allowed. When the coefficient of variation of the specimen was less than 10% [37], the test was considered to be effective. The test results for the unconfined compressive strengths of the parallel specimens with a 95% assurance rate were taken as the representative values.

**Table 11:** Results of the compaction tests on ER-OCSM specimens with different emulsified asphalt contents.

Test index	Emulsified asphalt content/%					
	1.0	2.0	3.0	4.0	5.0	6.0
Optimal water content *ω*_o_/%	7.67	7.97	8.12	8.09	8.24	8.20
maximum dry density *ρ*_max_/(g/cm^3^)	2.150	2.150	2.170	2.170	2.161	2.180

As shown in Table 10, when 3% cement was added, the recycled cement macadam under such a gradation reached the requirements for the unconfined compressive strength of the base (5–7 MPa) described in the technical regulations [30] under extremely heavy and extra-heavy traffic loads; this cement content was slightly lower than the typical cement consumption (according to the literature [36], the cement consumption of the high-grade highways in Jiangsu Province is generally between 3.5% and 4.5%; after 2010, ordinary Portland cement (PO42.5) has commonly been used, with a cement content of approximately 4%). These results indicate that step-by-step filling performs well for determining the dense skeleton gradation performance of recycled cement macadam.

### Influence of the emulsified asphalt content on the mechanical properties of ER-OCSM

#### Flexural tensile strength test

**Fig 10:**
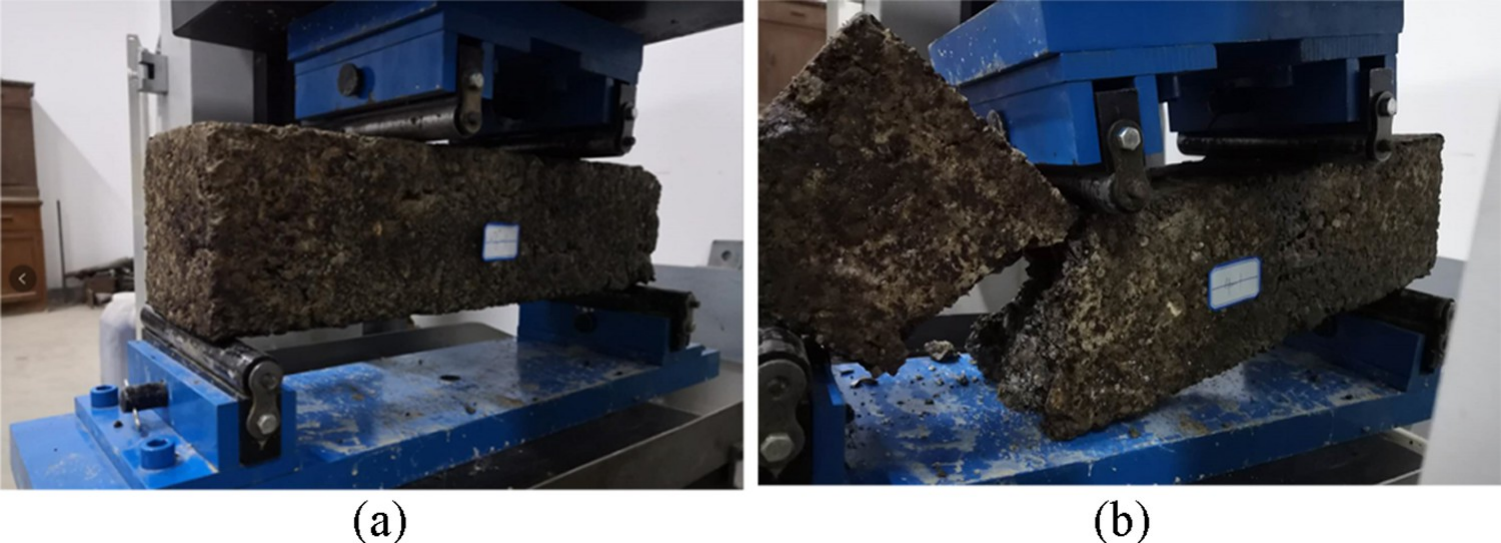
Flexural tensile strength testing for the ER-OCSM with a 4.0% emulsified asphalt content. (a) Before failure. (b) After failure.

Flexural tensile strength reflects the resistance ability of the base material to bending, which serves as an important index for pavement structure design. According to the requirements for flexural tensile strength tests specified in the standards [37], the specimens that reached the required age (90 d) were immersed for 24 h and then placed in the pressure test machine for flexural strength testing (Fig 10). The loading speed of the press machine was controlled at 50 mm/min, the maximum pressure P (N) of the specimen was recorded at the time of specimen damage, and the flexural strength of the ER-OCSM was calculated as follows:

$$R_{S}=\frac{PL}{b^{2}h}$$ (1)

where *R*_*s*_ is the flexural tensile strength (MPa), *P* is the failing load peak (N), *L* is the distance between the two supports (mm), *b* is the width of the specimen (mm) and *h* is the height of the specimen (mm).

Among the 12 parallel specimens of the same group for the flexural tensile strength test, most abnormal values were eliminated using the 3-fold mean squared error method, but 1 or 2 abnormal values were allowed. When the coefficient of variation of the specimen was less than 10%, the test was considered to be effective. The test results for the flexural tensile strengths of the parallel specimens with a 95% assurance rate were taken as the representative values.

**Fig 11:**
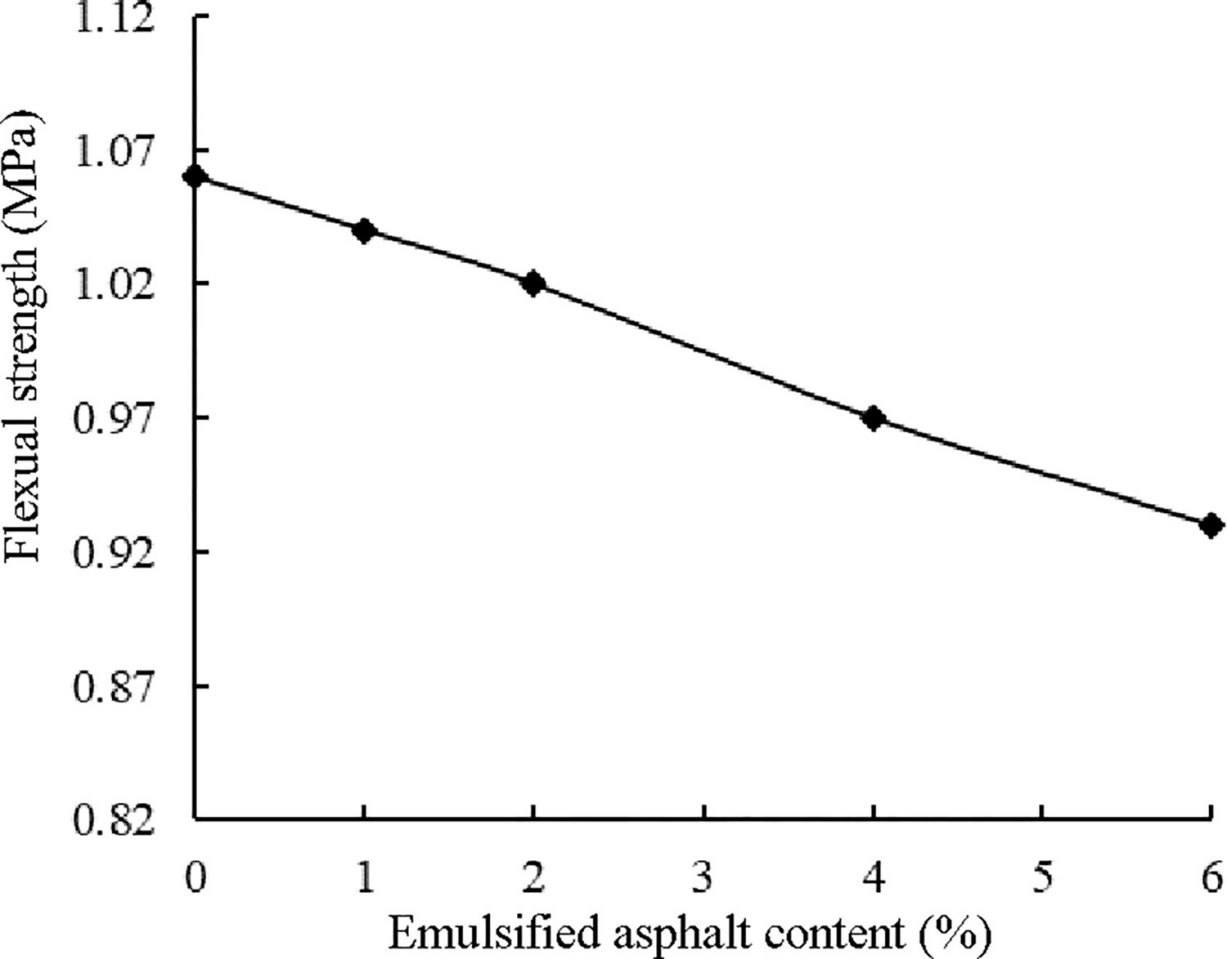
Changes in the flexural tensile strength.

The relationship between the flexural strength and the emulsified asphalt content based on the results in Table 12 is plotted in Fig 11.

**Table 12:** Test results for the flexural tensile strength of the ER-OCSM at an age of 90 d.

Emulsified asphalt content *ω*_r_/%	0.0	1.0	2.0	4.0	6.0
Flexural tensile strength *R*_S_/MPa	1.06	1.04	1.02	0.97	0.93

As shown in Fig 11, the flexural strength of the ER-OCSM decreased with increasing emulsified asphalt content (S1 File). The flexural strength of ER-OCSM with a 6.0% content was approximately 13% lower than that of ER-OCSM without emulsified asphalt; however, the flexural strengths of ER-OCSM specimens with emulsified asphalt contents of 1.0% and 2.0% were approximately 2% and 4% lower than that of ER-OCSM without emulsified asphalt. Our findings were in line with the change pattern of ER-OCSM splitting strength reported by Jiang et al. [41]; that is, with the increase in the added emulsified asphalt, the splitting strength of the mixture gradually decreased, and a specimen with a higher emulsified asphalt content demonstrated a greater decrease in splitting strength. Both splitting strength and flexural strength symbolize the tensile capacity of materials; therefore, it should be expected that the flexural tensile strength of ER-OCSM decreases gradually with the increase in emulsified asphalt content. Our findings indicate that the flexural strength of the ER-OCSM will decrease with the addition of emulsified asphalt. However, as long as the content of emulsified asphalt is controlled at a reasonable level, the influence of emulsified asphalt on the flexural strength is small.

For the cement-stabilized macadam material, both the flexural tensile strength and splitting strength characterize the tensile capacity of the material. However, the preparation of the beam specimens used in the flexural strength test was more complex than that of the column specimens used in the splitting strength test. Therefore, establishing the relationship between the flexural strength and splitting strength of the ER-OCSM will be helpful to calculate the flexural strength based on the splitting strength because the latter is easier to test. Based on the results of the ER-OCSM splitting strength tests conducted by Jiang et al. [41], the relationship between the flexural tensile strength and splitting strength of 90-d ER-OCSM was obtained (Table 13).

**Table 13:** Relationship between the flexural tensile strength and splitting strength of 90-d ER-OCSM.

Test index	Emulsified asphalt content/%				
	0.0	1.0	2.0	4.0	6.0
Flexural tensile strength *R*_S_/MPa	1.06	1.04	1.02	0.97	0.93
Splitting strength *R*_p_/MPa	0.80	0.66	0.65	0.51	0.48
Ratio between the flexural tensile strength and splitting strength	1.33	1.58	1.57	1.90	1.93

**Fig 12:**
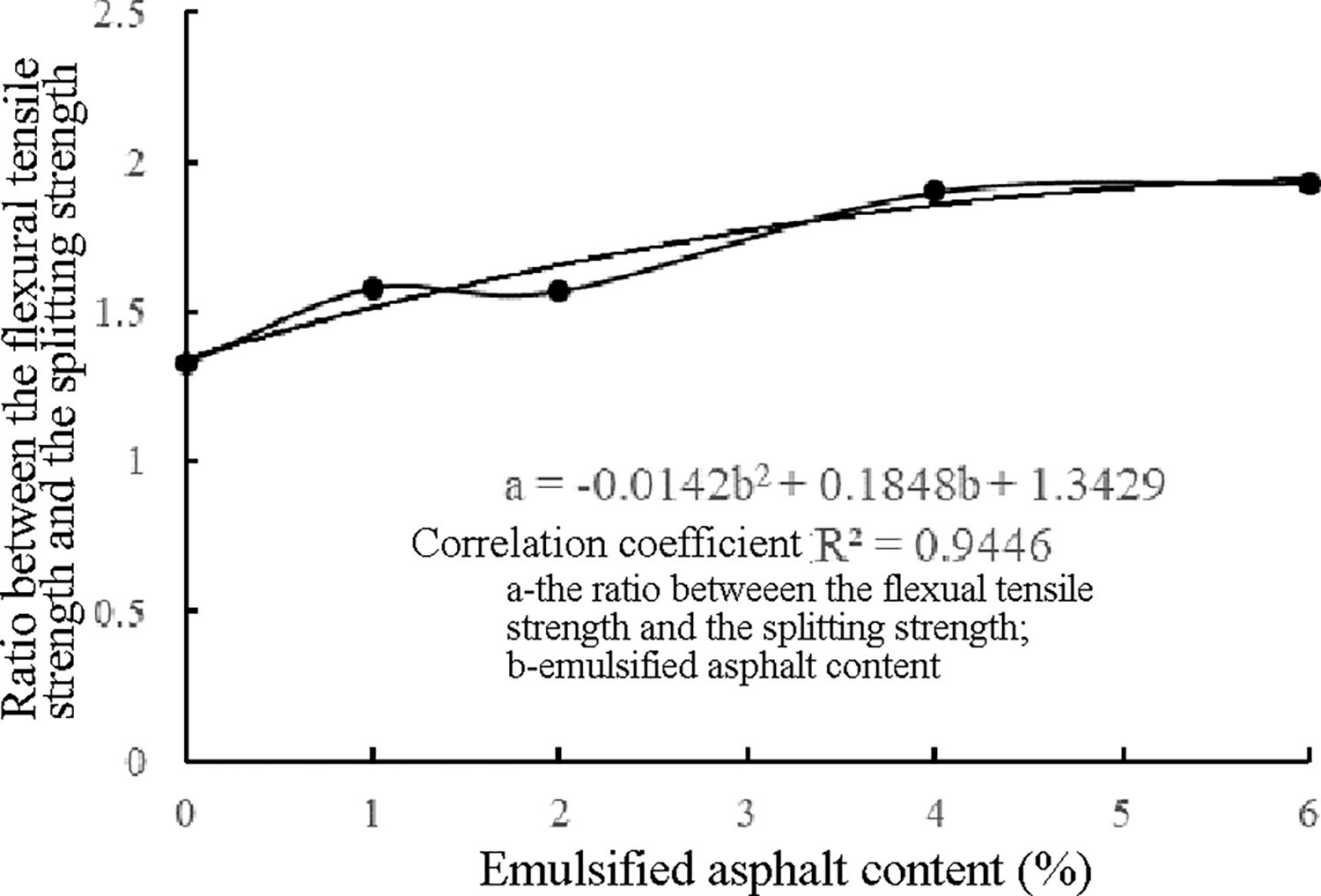
Relationship between the flexural tensile strength and splitting strength.

As shown in Table 13 and Fig 12, the flexural tensile strength of the ER-OCSM exhibited a good correlation with the splitting strength, which indicates that both indices express the tensile capacity of the material, just from different perspectives. As previously mentioned, in an actual test, the shaping process of a beam specimen used in the flexural tensile strength test is more complicated than that of a column specimen. The flexural tensile strength of ER-OCSM can also be calculated based on the splitting strength of a column specimen.

#### Dynamic elastic modulus test

The process of using traditional methods to determine the elastic modulus of cement-stabilized macadam materials is complex. Studies in recent years have adopted the resonant method and the acoustic method to determine the dynamic elastic modulus of cement-stabilized macadam materials to characterize the ability of materials to resist deformation, and satisfactory results have been achieved [42,43]. Therefore, in this study, the acoustic method was used to test the dynamic elastic modulus of the designed ER-OCSM. The dynamic elastic modulus of the ER-OCSM was calculated by the ZBL-U520A tester results of the propagation velocity of ultrasonic waves in the ER-OCSM specimen and the density of the specimen.

**Fig 13:**
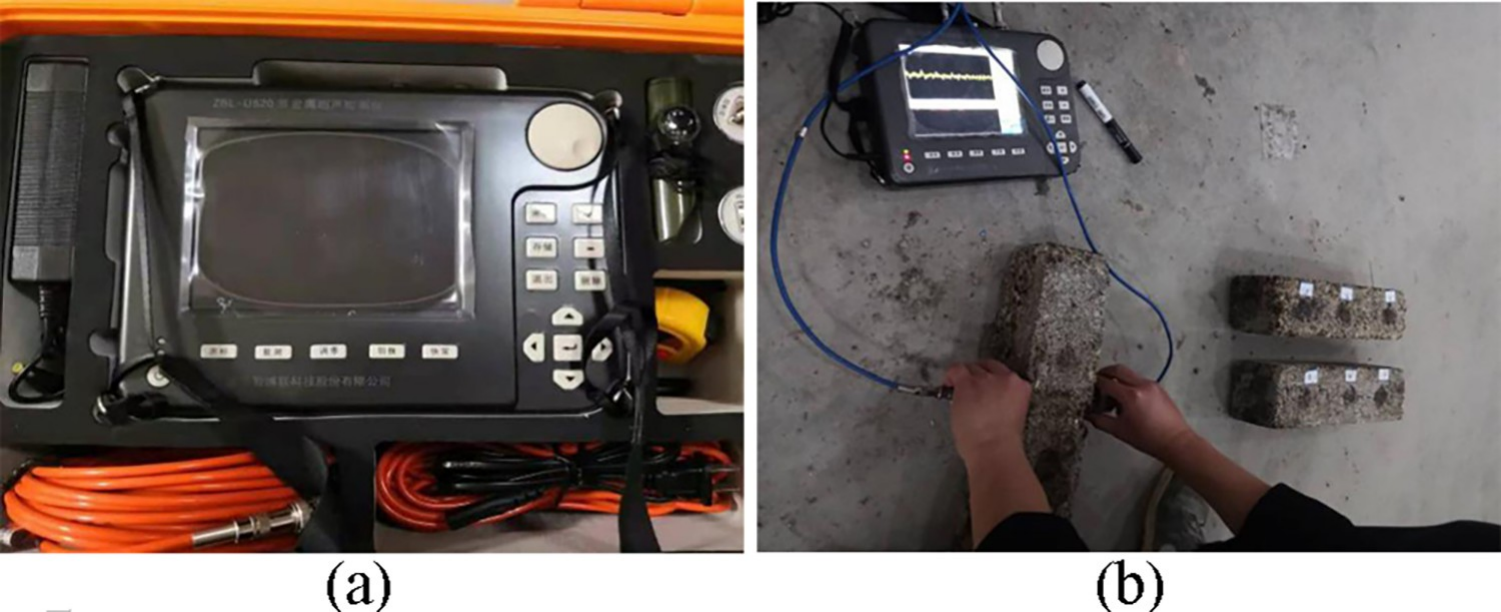
Dynamic elastic modulus test. (a) Nonmetal ultrasonic detector. (b) Dynamic elastic modulus testing process.

Before the test, the specimens were removed from the curing box after 7, 28, 60 and 90 d. The ZBL-U520A instrument was adjusted. After the two probes were coated with Vaseline, they were directed to the sides of the test piece. The waveform of the instrument screen was observed. After the waveform became stable, the wave speed transmitted within the specimen was read. In each group, 12 parallel specimens were prepared. Most abnormal values were eliminated using the 3-fold mean squared error method, but 1 or 2 abnormal values were allowed. When the coefficient of variation of the specimen was ≤15%, the test was considered to be effective. The test results for the dynamic elastic modulus of the parallel specimens with a 95% assurance rate were taken as the representative values. The testing process is shown in Fig 13.

The dynamic elastic modulus was calculated as follows:

$$E_{d}=\frac{\left(1+\mu )(1-2\mu \right)}{1-\mu }\times \rho \times V^{2}$$ (2)

where *E*_*d*_ is the dynamic elastic modulus (MPa), *ρ* is the density of the specimen (kg/m^3^), V is the ultrasonic wave speed (km/s), and *μ* is Poisson’s ratio, which was assumed to be 0.25 [44].

The dynamic elastic modulus results are summarized in Table 14.

**Table 14:** Results of the dynamic elastic modulus tests (unit, MPa).

Emulsified asphalt content/%	Age/d			
	7	28	60	90
0.0	14980	15608	15820	15957
1.0	14617	14796	15046	15348
2.0	14177	14376	14690	14911
4.0	13443	13825	14001	14091
6.0	12815	13185	13479	13796

**Fig 14:**
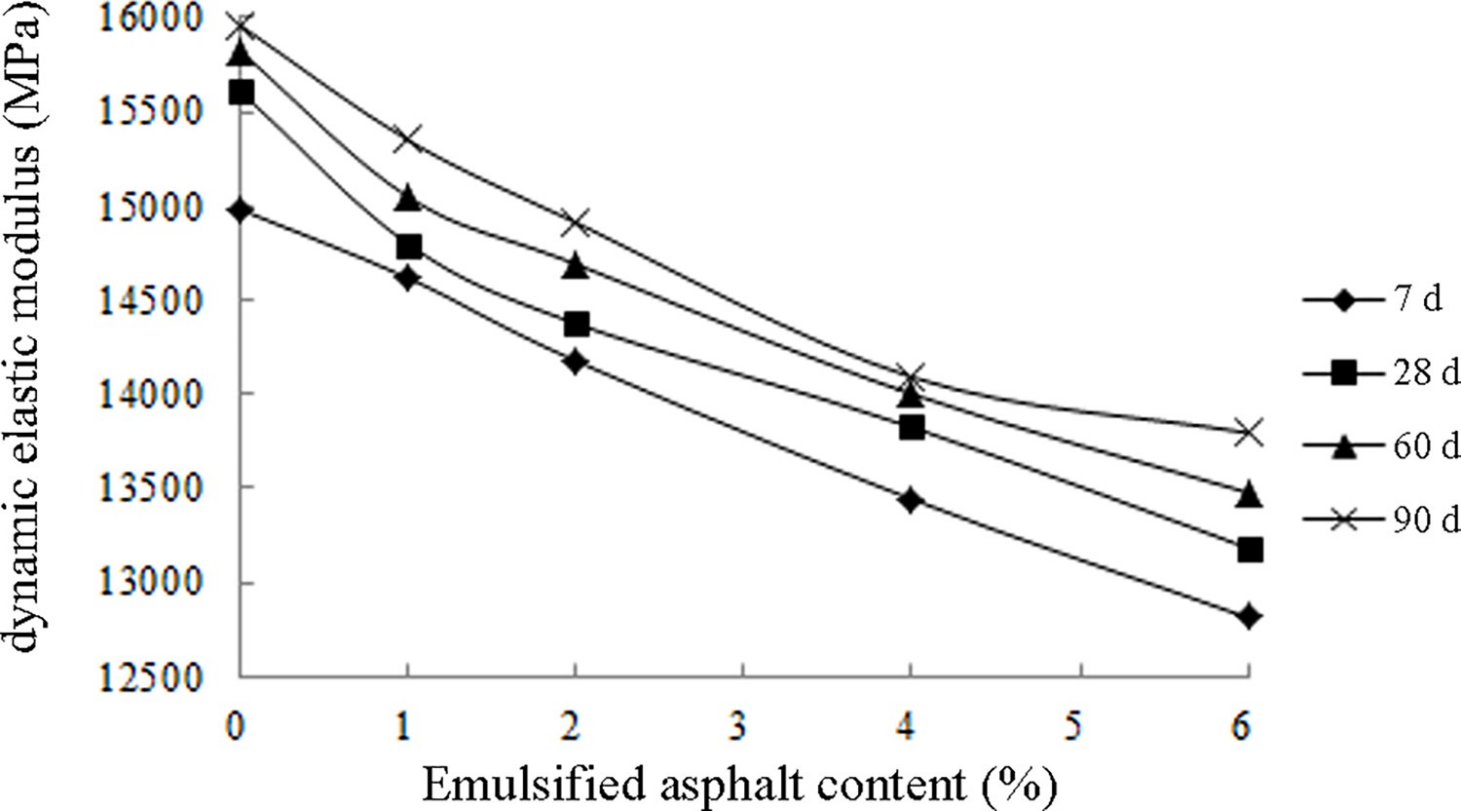
Dynamic elastic modulus results.

Fig 14 illustrates the results presented in Table 14. At the same age, the dynamic elastic modulus of the ER-OCSM decreased gradually with increasing emulsified asphalt content (S2 File). The dynamic elastic modulus of the 90-d ER-OCSM with an emulsified asphalt content of 6.0% was approximately 13.54% lower than that with an emulsified content of 0.0%.

Excessively large stiffness is one of the main factors that lead to cracking of the cement-stabilized base [36]. The addition of emulsified asphalt can effectively reduce the dynamic elastic modulus of the ER-OCSM base. Presumably, the underlying reason is as follows. With increasing age, due to a variety of physical and chemical reactions in the mixture, such as cement hydration, ion exchange and emulsified asphalt demulsification, the resulting products cause the mixture to form a complex and dense structure. The structure of the void space network of the asphalt inside a mixture enhances the deformation ability of the material; therefore, the elastic modulus gradually decreases with increasing emulsified asphalt content. This property is conducive to the uniform distribution of stress in the pavement structure and helps to improve the mechanical characteristics of the ER-OCSM base, thereby inhibiting or slowing the generation of base cracks and the formation of excessive reflection cracks.

The higher the content of emulsified asphalt is, the more noticeable the reduction effect of the elastic modulus. However, an excessive content of emulsified asphalt leads to an excessively reduced stiffness of the ER-OCSM base, which is not conducive to the road performance of the base. Therefore, controlling for the appropriate emulsified asphalt content allows the ER-OCSM base to not only become stronger but also retain a certain flexibility, thereby achieving the combined effect of rigidity and flexibility.

**Fig 15:**
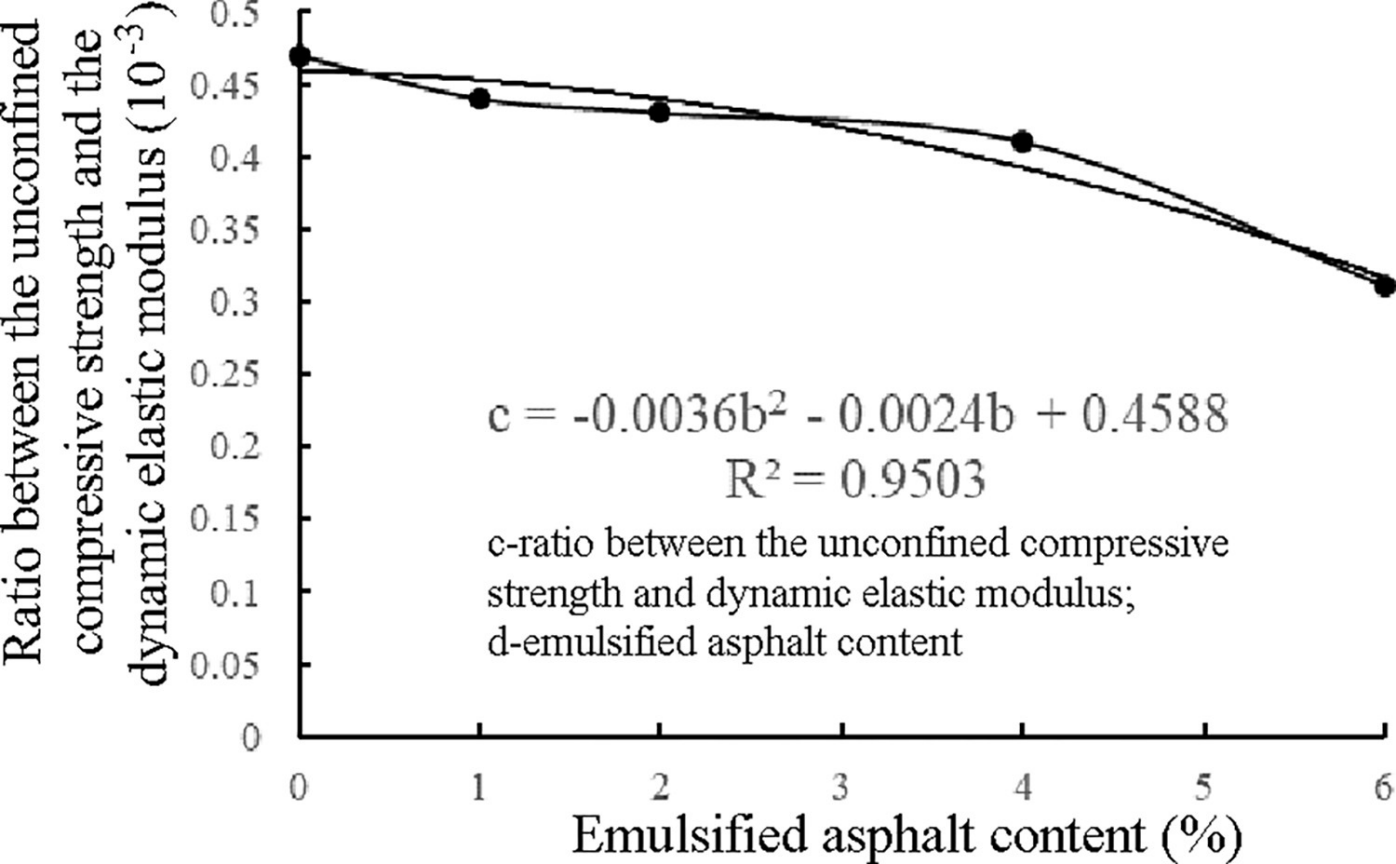
Relationship between the unconfined compressive strength and dynamic elastic modulus of the ER-OCSM.

In the design of the mix ratio of the semirigid base material, as well as in the construction stage, the unconfined compressive strength is the control index. However, in pavement structure design, the elastic modulus is the selected material design parameter. To quantitatively evaluate one index by another index, the relationship between the unconfined compressive strength and dynamic elastic modulus can be established. According to the test on the unconfined compressive strength of the ER-OCSM [42], the relationship between the unconfined compressive strength and dynamic elastic modulus of the 90-d ER-OCSM was obtained (Table 15 and Fig 15).

**Table 15:** Relationship between the unconfined compressive strength and dynamic elastic modulus.

Test index	Emulsified asphalt content%				
	0.0	1.0	2.0	4.0	6.0
Unconfined compressive strength *R*_f_/MPa	7.44	6.78	6.34	5.79	4.38
Dynamic elastic modulus *E*_d_/MPa	15957	15348	14911	14091	13976
Ratio between the unconfined compressive strength and dynamic elastic modulus/10^−3^	0.47	0.44	0.43	0.41	0.31

As shown in Fig 15, for the ER-OCSM specimens, the unconfined compressive strength exhibited a satisfactory correlation with the dynamic elastic modulus. During the recycling of pavement materials, the stiffness (modulus) index of the base material of the ER-OCSM can be calculated through the unconfined compressive strength, which is the construction quality control index. Thus, the deformation ability of the ER-OCSM base can be predicted, which is convenient for the design and analysis of the pavement structure layer.

### Influence of temperature on the mechanical properties of ER-OCSM

#### Influence of temperature on unconfined compressive strength

**Fig 16:**
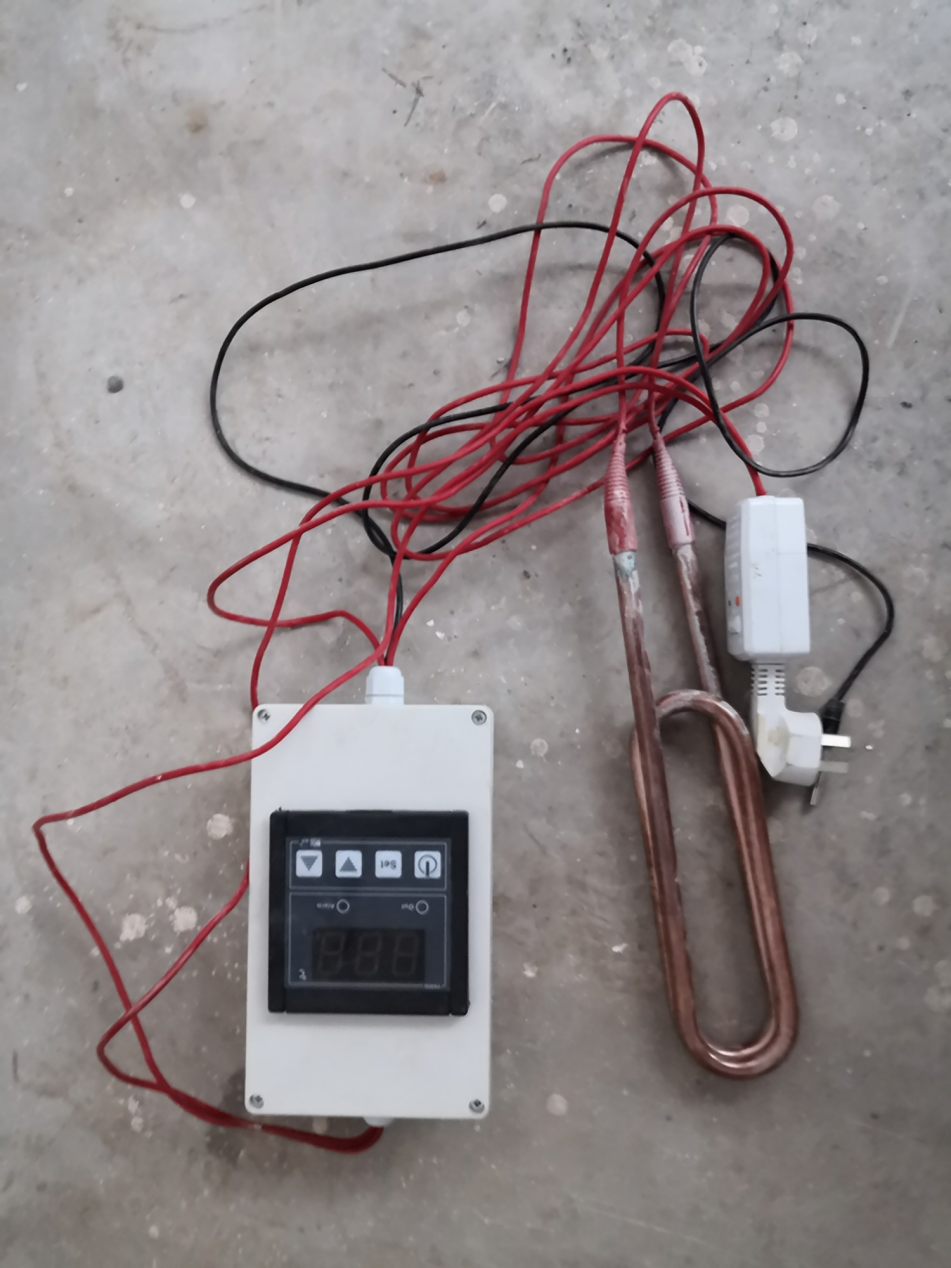
Custom heating and temperature control equipment.

The test was performed with heating and temperature control equipment made in-house using the constant-temperature water bath method (Fig 16). The water in the bath was heated to a predetermined temperature, and then a 7-d column specimen was put into the water bath, soaked for 24 h and removed for testing. The water bath temperatures were 5°C, 15°C, 25°C, 40°C, and 60°C.

The results of the unconfined compressive strength of the ER-OCSM at different temperatures are summarized in Table 16.

**Table 16:** Unconfined compressive strength of the ER-OCSM at different temperatures (unit, MPa).

Temperature/°C	Emulsified asphalt content/%				
	0.0	1.0	2.0	4.0	6.0
5	6.04	5.82	4.52	3.89	2.49
15	6.02	5.76	4.48	3.82	2.43
25	6.04	5.72	4.43	3.76	2.39
40	5.97	5.64	4.23	3.61	2.13
60	6.01	5.55	4.18	3.42	2.08

**Fig 17:**
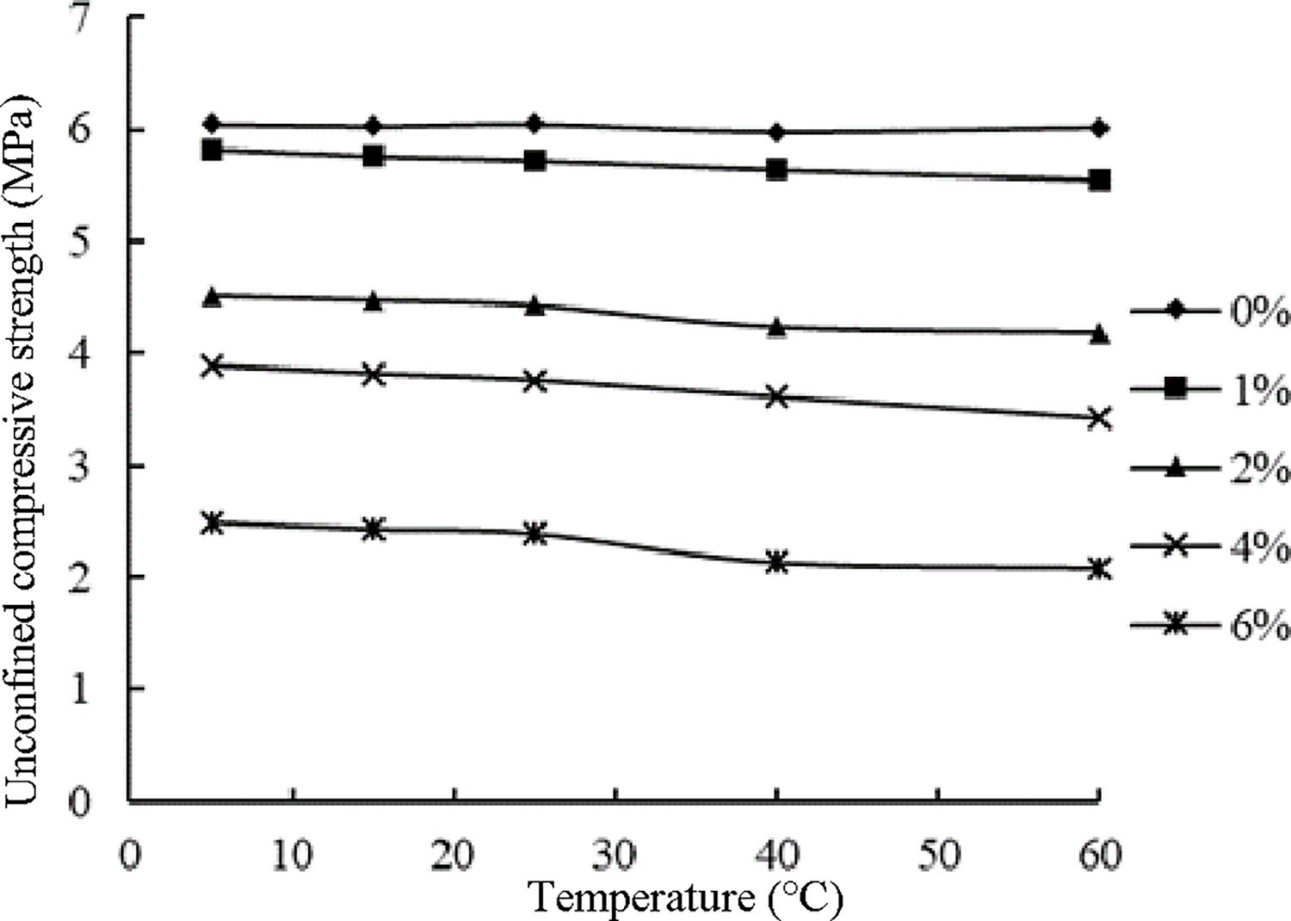
Unconfined compressive strength of the ER-OCSM at different temperatures.

Fig 17 illustrates the results presented in Table 16. As shown in Fig 17, with increasing temperature, the unconfined compressive strength of the specimens without emulsified asphalt exhibited no obvious change, while that of the specimens with emulsified asphalt decreased. The unconfined compressive strength of the specimens with a high emulsified asphalt content decreased greatly. The unconfined compressive strength of the specimens with a 4.0% emulsified asphalt content decreased by approximately 12.1%, and the unconfined compressive strength of the specimens with a 6.0% emulsified asphalt content decreased by approximately 16.5%. Zhang investigated the temperature sensitivity of asphalt pavement cold recycled mixtures and found that the unconfined compressive strength and splitting strength of cold recycled mixtures decreased with increasing temperature [29]. Fu et al. added emulsified asphalt to cement-stabilized macadam and found that after emulsified asphalt was added, the unconfined compressive strength and splitting strength of the mixture decreased, and the mixture also exhibited temperature sensitivity [38]. Presumably, with increasing temperature, the viscosity of the asphalt film covering the aggregates in the mixture decreased, which weakened the bonding performance of the material. Therefore, for an ER-OCSM specimen with a higher emulsified asphalt content, the decrease in its unconfined compressive strength became more noticeable with the increase in temperature.

#### The influence of temperature on the flexural tensile strength of ER-OCSM

The specimen temperature was again controlled using the constant-temperature water bath method. A beam specimen aged for 90 d was soaked in water for 24 h. The surface water was removed, and the specimen was dried for the flexural tensile strength test. The test results are summarized in Table 17.

**Table 17:** Flexural tensile strength of the tested ER-OCSM at different temperatures (unit, MPa).

Temperature/°C	Emulsified asphalt content/%				
	0.0	1.0	2.0	4.0	6.0
5	1.07	1.05	1.04	0.97	0.94
15	1.05	1.03	1.02	0.96	0.93
25	1.05	1.02	1.01	0.96	0.91
40	1.06	1.02	0.96	0.91	0.84
60	1.04	0.98	0.92	0.87	0.81

**Fig 18:**
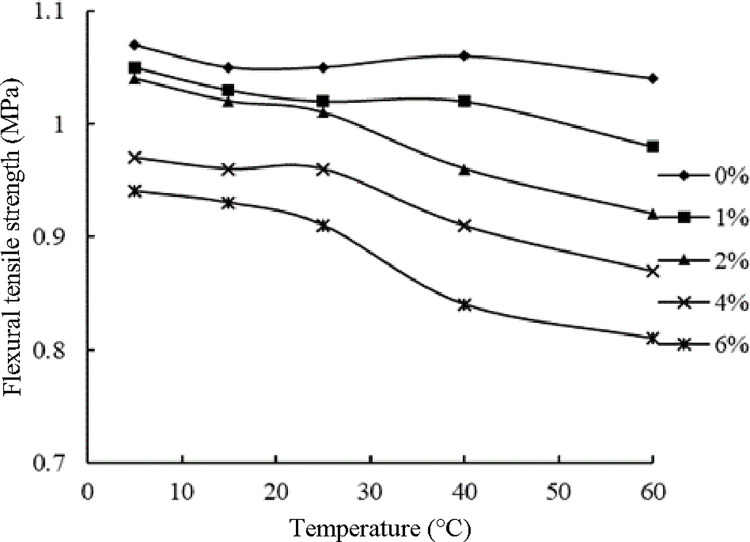
Flexural tensile strength of the ER-OCSM at different temperatures.

The results in Fig 18 are plotted based on the values in Table 17. As shown in Fig 18, with increasing temperature, in the low-temperature range (5–25°C), the flexural tensile strength of the ER-OCSM slightly decreased. At 25°C, the specimen with a 6.0% emulsified asphalt content showed the greatest decrease in flexural tensile strength (approximately 3.19%), and the average decrease in flexural tensile strength observed for all specimens was approximately 2.37% from that observed at 5°C. In contrast, within the high-temperature range (25–60°C), noticeable changes were observed, and the average decrease of all specimens was 9.3% at 60°C from that at 5°C. Among these specimens, the ER-OCSM with a 6% emulsified asphalt content showed the greatest decrease, which was approximately 13.83%.

The possible reasons for this behavior are discussed as follows. On the one hand, the temperature increased, and the viscosity of the asphalt film decreased, which affected the flexural tensile strength of the material. On the other hand, during the flexural tensile strength testing, a loading rate of 50 mm/min was adopted, according to the standards, which led to the test piece being crushed quickly, and the failure speed was too fast to determine the sensitivity of the material to temperature. Therefore, within the low-temperature range, the flexural strength of the ER-OCSM specimens with emulsified asphalt was less affected by temperature. However, with increasing temperature, the viscosity of the asphalt film decreased noticeably, and the decrease in the flexural strength of the ER-OCSM specimens became noticeable within the high-temperature range. These findings also indicate that the content of emulsified asphalt in ER-OCSM should not be too high; otherwise, the sensitivity of the material to the temperature will be insufficient to achieve a good flexural tensile performance.

#### Influence of temperature on the dynamic elastic modulus of the ER-OCSM

The acoustic method was used to test the influence of temperature on the dynamic elastic modulus of the ER-OCSM. Because the moisture content will affect the propagation speed of ultrasonication in the specimen and then affect the test results, the dynamic elastic modulus of the ER-OCSM specimens at 5°C, 15°C, 25°C, 40°C and 60°C were tested. The test results are shown in Table 18.

**Table 18:** Dynamic elastic modulus of the ER-OCSM at different temperatures (unit, MPa).

Temperature/°C	Emulsified asphalt content/%				
	0	1	2	4	6
5	14088	13297	12989	12562	12461
15	14042	13071	12535	12379	12165
25	14053	12715	12319	12135	11368
40	14045	12478	12022	11893	10904
60	13939	12240	11420	11040	10459

**Fig 19:**
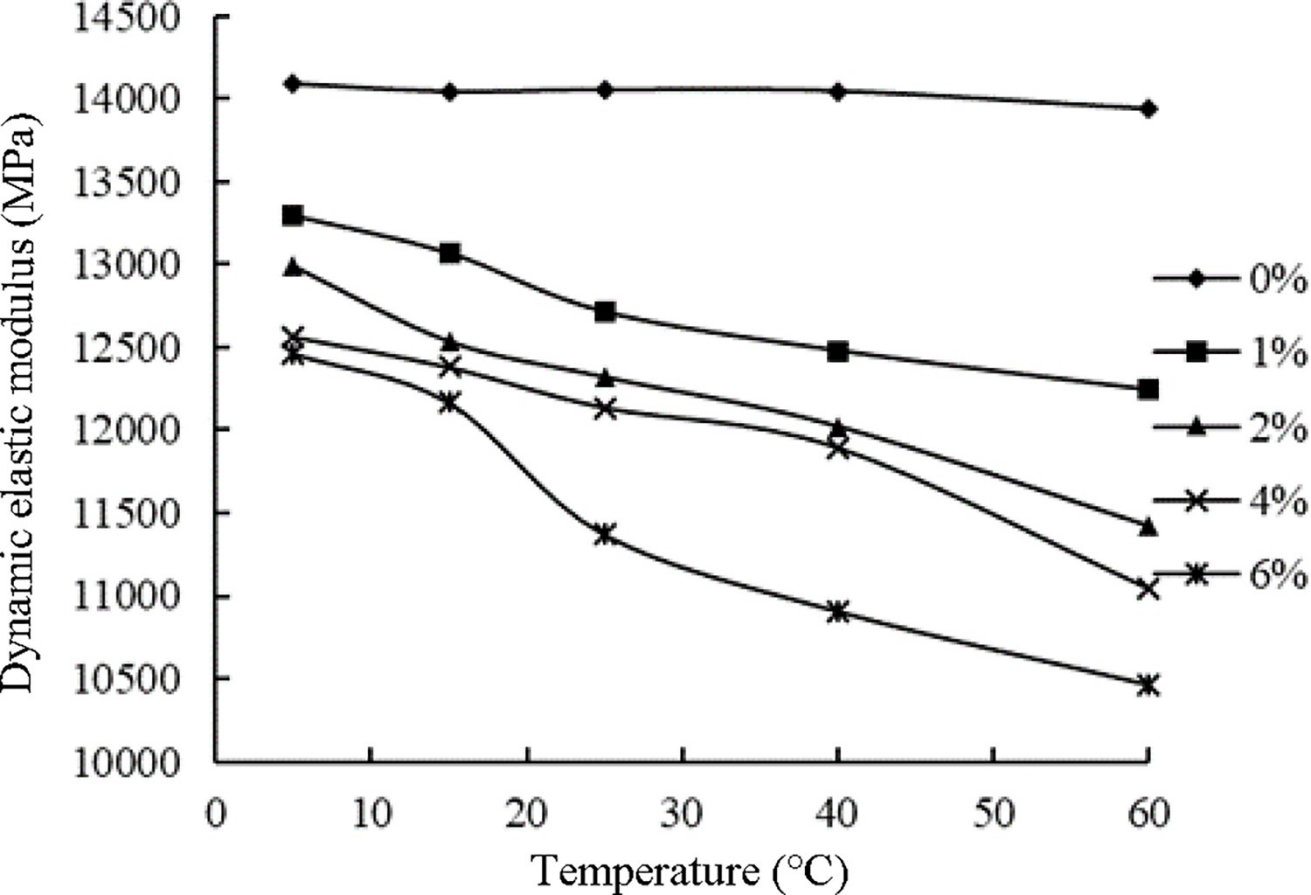
Dynamic elastic modulus of the ER-OCSM at different temperatures.

Fig 19 illustrates the results presented in Table 18. As shown in Fig 19, the dynamic elastic moduli of the specimens without emulsified asphalt showed little difference at different temperatures. With the addition of emulsified asphalt, the specimens showed temperature sensitivity. With increasing temperature, the dynamic elastic modulus of the ER-OCSM decreased gradually. The higher the emulsified asphalt content was, the more noticeable the decrease in the dynamic elastic modulus. The reasons for these findings may be as follows. With increasing temperature, especially when the temperature is above 40°C, the dynamic elastic modulus of the specimen decreases regardless of whether emulsified asphalt is added. In the high-temperature range, when the emulsified asphalt content is high, the viscosity of the asphalt film structure formed in the mixture decreases, which reduces the integrity of the material, thereby resulting in a decrease in the test wave velocity and dynamic elastic modulus.

## Conclusions

Cement and emulsified asphalt have been used to regenerate pavement materials. However, most of the previous research focused on the mixture of waste asphalt pavement materials with base materials, the asphalt pavement materials or the mixture of waste asphalt pavement materials with recycled base materials and new materials. In this study, the aggregate was 100% waste cement-stabilized macadam base milling material, which determined that the composition and physical characteristics of our mixture material differed to some extent from those reported in the literature. In addition, the findings of our study have a higher environmental significance. Furthermore, because the ER-OCSM contains asphaltene from emulsified asphalt and the mixture has a certain temperature sensitivity, the temperature sensitivity of the recycled mixture was investigated in this study, which has rarely been reported in the research of similar materials.

The main conclusions of this study are as follows:

1. After sieving, the OCSM milling material shows that the contents of particles with sizes of 19–9.5 mm and 9.5–4.75 mm were relatively high. Under natural gradation, these contents were beyond the standard range. Based on the step-by-step filling test and according to the characteristics of different aggregates, an appropriate test method was selected to achieve a uniform and dense state, given the characteristics of different aggregates (that is, the coarse aggregates were tamped and the fine aggregates were premixed and tamped), and a design method was established for OCSM gradation to achieve a dense skeleton. The results showed that the design gradations were basically within the range specified in the standards, with the only exception being that the contents of the 9.5–4.75 mm aggregates were slightly higher than the specified upper limit.
2. Because the surfaces of the recycled coarse aggregates were mostly covered by cement mortar, the surfaces were rough, which was conducive to improving the adhesion between the recycled coarse aggregates and cement. The crushing value was the only index obtained that was higher than that specified in the standards.
3. At the same age and temperature, the flexural strength and dynamic elastic modulus of the ER-OCSM specimens decreased gradually with increasing emulsified asphalt content, and the higher the emulsified asphalt content was, the more obvious the decrease.
4. The emulsified asphalt content and age had a great influence on the flexural strength and dynamic elastic modulus of the ER-OCSM. The flexural strength of the ER-OCSM decreased with increasing emulsified asphalt content. With the same emulsified asphalt content, the dynamic elastic modulus of the ER-OCSM increased gradually with increasing age. At the same age, the dynamic elastic modulus of the ER-OCSM decreased with increasing emulsified asphalt content.
5. Good correlations were observed between the flexural strength and splitting strength of the ER-OCSM specimens.
6. ER-OCSM has temperature-sensitive characteristics, that is, it is highly sensitive to temperature. An increase in temperature decreased the viscosity of the asphalt in the mixture. The unconfined compressive strength, flexural strength and dynamic elastic modulus all decreased with increasing temperature. In the low-temperature range (5–25°C), the decrease was small, and the difference in each content was small (the maximum difference was approximately 2.16%). The average rate of decrease was 2.37%. However, the changes were noticeable within the high-temperature range (25–60°C). The higher the emulsified asphalt content was, the more obvious the decrease rate.
7. The addition of emulsified asphalt can reduce the rigidity of ER-OCSM. However, the content of emulsified asphalt should be controlled. Too much emulsified asphalt will reduce the mechanical properties of the materials, which will adversely affect the comprehensive performance of the road.

The results of this study may provide some reference for the recycling of waste cement-stabilized macadam bases and may also provide a reference for the emulsification and recycling of similar semirigid base materials. However, this study had some limitations, which also constitute future research directions based on the results of this study. First, the fatigue performance, frost resistance and other road performance factors of ER-OCSM material were not investigated, and future studies need to take these factors into consideration. Second, the chemical composition of the milling material was not analyzed in this study, which is a worthy research direction in the future. This type of study may be helpful in clarifying the relationship between milling materials and binders from the perspective of structural morphology.

## Supporting information

S1 File Source data of the ER-OCSM flexural tensile strength test. (XLSX)

S2 File Source data of the dynamic elastic modulus test of ER-OCSM with different emulsified asphalt contents. (XLSX)
